# Cytotoxic and Antioxidant Compounds from the Stem Bark of *Goniothalamus tapisoides* Mat Salleh

**DOI:** 10.3390/molecules18010128

**Published:** 2012-12-21

**Authors:** Rosalind Pei Theng Kim, Vicky Bihud, Khalit bin Mohamad, Kok Hoong Leong, Jamaludin bin Mohamad, Fasihuddin bin Ahmad, Hazrina Hazni, Noraini Kasim, Siti Nadiah Abdul Halim, Khalijah Awang

**Affiliations:** 1 Department of Chemistry, University of Malaya, 50603 Kuala Lumpur, Malaysia; Email: rosalindkim@hotmail.com (R.P.T.K.); hazrinahazni@um.edu.my (H.H.); nadiahhalim@um.edu.my (S.N.A.H.); 2 Faculty of Applied Sciences, MARA University of Technology, 40450 Shah Alam, Selangor, Malaysia; Email: vicky@salam.uitm.edu.my (V.B.); norainikasim@salamuitm.edu.my (N.K.); 3 Department of Pharmacy, University of Malaya, 50603 Kuala Lumpur, Malaysia; Email: khalitmohamad@um.edu.my (K.M.); leongkh@um.edu.my (K.H.L.); 4 Institute of Biological Sciences, Faculty of Sciences Building, University of Malaya, 50603 Kuala Lumpur, Malaysia; Email: jamal@um.edu.my; 5 Faculty of Resource Science and Technology, Universiti Malaysia Sarawak, 94300 Kota Samarahan, Sarawak, Malaysia; Email: bfasih@frst.unimas.my

**Keywords:** *Goniothalamus tapisoides*, goniomicins A–D, tapisoidin, cytotoxicity, antioxidant activity

## Abstract

Eleven compounds: goniomicin A (**1**), goniomicin B (**2**), goniomicin C (**3**), goniomicin D (**4**), tapisoidin (**5**), goniothalamin (**6**), 9-deoxygoniopypyrone (**7**), pterodondiol (**8**), liriodenine (**9**), benzamide (**10**) and cinnamic acid (**11**), were isolated from the stem bark of *Goniothalamus tapisoides*. All compounds were identified by spectroscopic analysis and, for known compounds, by comparison with published data. Goniothalamin (**6**) exhibited mild cytotoxic activity towards a colon cancer cell line (HT-29), with an IC_50_ value of 64.17 ± 5.60 µM. Goniomicin B (**2**) give the highest antioxidant activity in the DPPH assay among all compounds tested, with an IC_50_ of 0.207 µM.

## 1. Introduction

The genus *Goniothalamus* (Annonaceae) comprises about 160 species of shrubs and trees native to tropical and subtropical Asia [1]. *Goniothalamus tapisoides* Mat Salleh, locally known as “selada” or “semukau”, is endemic to Borneo, especially the Southern part of Sarawak. This species is a small tree of about 5 m in height and is used by the native folks as an abortifacient and to cure poisonous animal bites such as snake, scorpion or insect bites. It is also used to relieve stomachaches [2].

In recent years, *Goniothalamus* species have been receiving considerable attention because of their reputation for producing styryl lactones and acetogenins which possessre markable cytotoxic and antitumor properties against various human tumor cell lines such as A-549 (lung carcinoma), HL-60 (promyelocytic leukemia), and SGC-7901 (stomach cancer) [1,3]. Previous studies on the mechanism of action of (*R*)-goniothalamin have shown that it is able to induce apoptosis in MCF-7 (breast cancer) and HL-60 human cancer cells [3,4,5,6].

**Figure 1:**
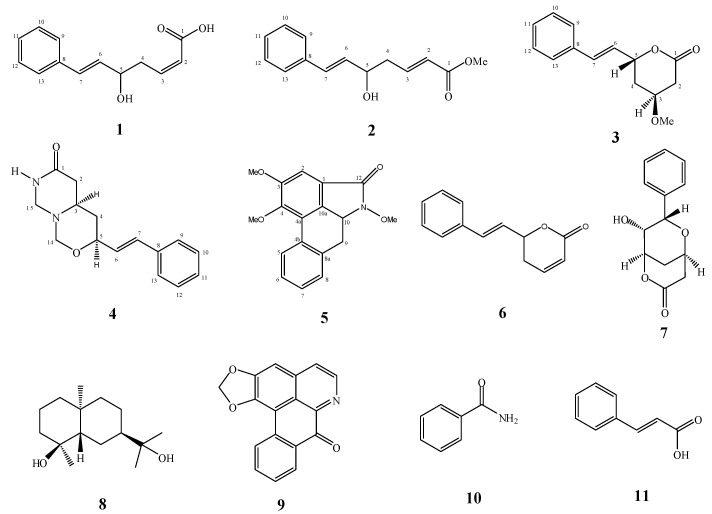
Structure of compounds **1**–**11**.

In our continuous effort to search for new and bioactive compounds from the Malaysian flora [7,8,9,10], we have embarked on a chemical study of the cytotoxic stem bark extracts of *G. tapisoides* Mat Salleh*.* To the knowledge of the authors, such a phytochemical study of this planthas never been reported. From this study, the eleven compounds shown in Figure 1 were isolated, of which five are new: goniomicin A (**1**), goniomicin B (**2**), goniomicin C (**3**), goniomicin D (**4**) and tapisoidin (**5**). The known compounds are goniothalamin (**6**), 9-deoxygoniopypyrone (**7**), pterodondiol (**8**), liriodenine (**9**), benzamide (**10**) and cinnamic acid (**11**). Compounds **1**–**6** were tested for cytotoxic activity against eight cancer cell lines and for antioxidant activity.

## 2. Results and Discussion

### 2.1. Isolation and Chemistry

Goniomicin A (**1**) was obtained as a pale yellowish amorphous powder, 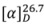
+2.5° (c 0.0239, MeOH). A molecular formula of C_13_H_14_O_3_ was deduced from the ESI-TOF-MS spectrum, that showed a strong fragmentation peak at *m/z* 199.1020 [M−H−H_2_O]^−^ (calc. 199.0759) corresponding to the loss of a water molecule, thus indicating the presence of a hydroxyl group. This is in agreement with the ^13^C-NMR and HSQC spectra which confirmed the presence of thirteen carbons. The UV spectrum (λ_max_ 206 and 251 nm) suggested the presence of a phenyl group [11]. The IR spectrum showed absorptions of hydroxyl (ν_max_ 3344 cm^−1^) and carbonyl (ν_max_ 1668 cm^−1^) functionalities. 

The ^1^H-NMR spectrum (Table 1) revealed downfield signals at δ 5.96, δ 6.12, δ 6.20 and δ 6.59 assignable to H-2, H-3, H-6 and H-7, respectively, thereforeindicating the presence of four olefinic protons. The coupling constant between H-2 and H-3 is 11.5 Hz which is indicative of a *cis-* configuration [11]. On the other hand, H-6 and H-7 assumed a *trans*-configuration, with a *J* coupling value of 16.0 Hz.

**Table 1:** ^1^H-NMR, ^13^C-NMR and HMBC (400 MHz) data of **1** and **2** (CDCl_3_, δ in ppm, *J* in Hz).

Atom	1			2		
No.	δ ^13^C	δ ^1^H	HMBC	δ ^13^C	δ ^1^H	HMBC
1	169.6	-		166.8	-	
2	125.3	5.96 (1H, *d*, *J* = 11.5)	C-1, C-3, C-4	123.9	5.94 (1H, *d*, *J* = 15.6)	C-1, C-4
3	140.6	6.12 (1H, *dt*, *J* = 8.6, 11.5)	C-1, C-4, C-5	144.7	7.00 (1H, *m*)	C-1, C-4
4α	36.6	2.76 (1H, *m*)	C-2, C-3, C-5, C-6	40.2	2.54 (2H, *m*)	C-2, C-3, C-5, C-6
4β		2.81 (1H, *m*)				
5	71.5	4.41 (1H, *m*)	C-3, C-4, C-7	71.6	4.44 (1H, *dd*, *J* = 6.6, 13.3)	C-3, C-7
6	131.9	6.20 (1H, *dd*, *J* = 16.0, 6.7)	C-4, C-5, C-8	130.9	6.22 (1H, *dd*, *J* = 16.0, 6.6)	C-5, C-8
7	129.9	6.59 (1H, *d*, *J* = 16.0)	C-5, C-8, C-9, C-13	131.3	6.61 (1H, *d*, *J* = 16.0)	C-5, C-9, C-13
8	136.7	-		136.3	-	
9,13	126.5	7.19–7.34 (*m*)		126.5	7.24–7.38 (*m*)	
10,12	128.6	7.19–7.34 (*m*)	C-8	128.6	7.24–7.38 (*m*)	C-8
11	127.6	7.19–7.34 (*m*)		128.0	7.24–7.38 (*m*)	
1-OH	-	6.27 (OH, *br s*)	C-2	-	-	
5-OH	-	6.57 (OH, *br s*)	C-5	-	-	
1-OMe	-	-		51.6	3.72 (3H, *s*)	C-1

The ^13^C-NMR spectrum (Table 1) showed the presence of one methylene, ten methines, one quaternary carbon and one carbonyl. The carbonyl carbon (C-1) resonated at δ 169.6. The adjacent C-2 and C-3 carbons gave signals at δ 125.3 and δ 140.6, respectively. The downfield shift of C-3 is due to the resonance effect of the α-β unsaturated carboxylic acid moiety [11]. In addition, a deshielded oxymethine signal attributable to C-5 was apparent at δ 71.5.

**Scheme 1:**
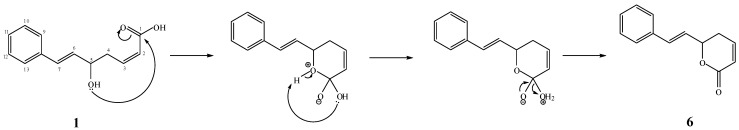
Dehydration and cyclization of **1** to **6**.

The ^1^H-^1^H COSY spectrum and ^1^H-^13^C HSQC spectrum confirmed the connectivities between C-2–C-3–C-4–C-5–C-6–C-7. The HMBC correlations indicated the linkage of the C-2 to carbonyl C-1 and C-7 to the phenyl ring C-8 (Table 1). Thus, the structure of goniomicin A (**1**) was elucidated as illustrated in Figure 1. The occurrence of compound **1** in *Goniothalamus tapisoides* is of interest since it may be a precursor of goniothalamin (**6**). A plausible biogenetic pathway for the formation of **6** from **1** is illustrated in Scheme 1. Compound **1** undergoes a dehydration and cyclization reaction to form **6**.

Goniomicin B (**2**) was isolated as pale yellowish amorphous powder, 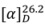
+2.4° (c 0.0082, CH_2_Cl_2_). Its molecular formula, C_14_H_16_O_3_, was deduced from the ESI-TOF-MS (*m/z* 231.1201, [M−H]^−^; calc. 231.1021). The IR spectrum showed a strong absorptionfora conjugated carbonyl group of an ester at 1718 cm^−1^, while the UV absorption bands at 207 and 251 nmindicated the presence of a phenyl group [11].

The ^1^H-NMR and ^13^C-NMR spectra of **1** and **2** (Table 1) are very similar to each other, except for the existence of a three proton singlet at δ 3.72 and a methyl carbon at δ 51.6 in the ^1^H- and ^13^C-NMR spectra, respectively. This observation suggested the presence of an additional methoxyl group, which is attached to the C-1 carbonyl group. A noticeable difference was also observed in the coupling constant value (15.6 Hz) of protons H-2 and H-3, suggesting a *trans*-configuration. From the ^1^H-NMR, ^13^C-NMR, ^1^H–^1^H COSY, HSQC and HMBC spectra analyses, the complete assignment ofgoniomicin B (**2**) was established.

Goniomicin C (**3**) was obtained as pale yellowish amorphous powder, 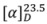
−4.9° (c 0.0122, CH_2_Cl_2_). The ESI-TOF-MS spectrum gave a prominent peak at *m/z* 231.1077, [M−H]^−^; (calc. 231.1021), corresponding to the molecular formula C_14_H_16_O_3_. The IR spectrum showed a C=O stretching absorption bands at 1731 cm^−1^ and C-O stretching ones at 1241 and 1090 cm^−1^. The UV absorptions at 206 and 251 nmsuggested the existence of a phenyl group [11].

The ^1^H-NMR spectrumof **3** is relatively similar to that of goniothalamin (**6**). However, the olefinic proton signals of the lactone ring are absent. Instead two methylene proton signals and one oxymethine proton signal appeared at δ 2.73 and δ 3.82, corresponding to H_2_-2 and H-3, respectively. Another oxymethine signal, assignable to H-5, appeared at δ 5.20 as a *ddd* with coupling constants of 11.0, 6.4 and 3.5 Hz respectively (Table 2). In addition, one singlet corresponding to three protons of a methoxyl group was apparent at δ 3.36. The ^13^C-NMR spectrum showed the expected fourteen carbons: one methyl, two methylene, nine methines and two quaternary carbons. The oxymethine carbons, C-3 and C-5, resonated at δ 71.4 and δ 76.2, respectively. In the HMBC spectrum (Table 2), the carbonyl carbon (C-1) signal (δ 169.7) correlated with the protons signal at δ 2.73 (H_2_-2), while the carbon at δ 71.4 (C-3) correlated with the proton at δ 5.20 (H-5). Thus it can be deduced that the methoxy group is attached to C-3.

**Table 2:** ^1^H-NMR, ^13^C-NMR and HMBC (400 MHz) data of **3** (CDCl_3_, δ in ppm, *J* in Hz).

Atom no.	δ ^13^C	δ ^1^H	HMBC	Atom no.	δ ^13^C	δ ^1^H	HMBC
1	169.7	-		7	132.5	6.68 (1H, *d*, *J* = 16.0)	C-5, C-9, C-13
2	35.7	2.73 (2H, *td*, *J* = 4.3, 1.4)	C-1	8	136.0	-	
3	71.4	3.82 (1H, *m*)		9,13	126.7	7.24–7.37 (*m*)	
4α	33.7	1.87 (1H, *ddd*, *J* = 14.8, 11.0, 3.5)		10,12	128.8	7.24–7.37 (*m*)	
4β		2.18 (1H, *m*)		11	128.3	7.24–7.37 (*m*)	
5	76.2	5.20 (1H, *ddd*, *J* = 11.0, 6.4, 3.5)	C-3	3-OMe	56.3	3.36 (3H, *s*)	C-3
6	126.6	6.18 (1H, *dd*, *J* = 16.0, 6.4)	C-5, C-8				

The relative stereochemistry of **3** was established by the NOESY spectrum. H-5α, which is axially oriented, correlated with H-4, therefore suggesting that H-4 adopts an α spatial orientation [12]. H-6 showed correlation to H-5β thus implyingthat H-6 is β oriented.With the aid of the ^1^H-NMR, ^13^C-NMR, ^1^H-^1^H COSY, HSQC and HMBC spectra analyses, the full assignment of goniomicin C (**3**) was determined.

Goniomicin D (**4**) was crystalisedfrom a solution of hexane-dicholoromethane as white rod crystals (mp. 240–243 °C), 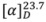
+37.17° (c 0.0191, MeOH). Its molecular formula was determined to be C_15_H_18_O_2_N_2_ by ESI-TOF-MS (*m/z* 281.1278, [M+Na]^+^; calc. 281.1266). The IR spectrum showed absorption peaks of N-H stretching at 3413.15 cm^−1^ and a C=O group at 1662 cm^−1^ [11]. The UV spectrum revealed maxima at 206 and 252 nm, suggesting the presence of a phenyl group [11]. 

The ^1^H-NMR spectrum showed four methylene signals at δ 2.28/2.64 (H_2_-2), δ 1.74 (H_2_-4), δ 4.13/4.64 (H_2_-14) and δ 3.82/4.34 (H_2_-15) (Table 3). The downfield shift of H_2_-14 and H_2_-15 were due to the neighboring nitrogen and oxygen. The ^13^C-NMR showed signals corresponding to fifteen carbons: four methylenes, nine methines, one quaternary and one carbonyl carbon. The methylene carbons C-14 and C-15 resonated downfield at δ 82.9 and δ 58.9, respectively. The carbonyl carbon (C-1) gave a peak at δ 168.9. The HMBC spectrum (Figure 1) revealed cross peaks betweenthe methylene at δ 4.13/4.64 (H_2_-14) and C-3 (δ 54.1), C-5 (δ 77.3) and C-15 (δ 58.9). Two methylene signals of H_2_-2 (δ 2.28/2.64) and H_2_-15 (δ 3.82/4.34) were correlated to the carbonyl C-1. From a ^1^H–^1^H COSY experiment, the entire sequence C-2–C-3–C-4–C-5–C-6 was identified.Thorough analyses of 1D and 2D NMR data analyses allowed the full assignment of all protons and carbons as listed in Table 3.

**Table 3:** ^1^H-NMR, ^13^C-NMR and HMBC (400 MHz) data of **4** (CDCl_3_, δ in ppm, *J* in Hz).

Atom no.	δ ^13^C	δ ^1^H	HMBC	Atom no.	δ ^13^C	δ ^1^H	HMBC
1	168.9	-		9,13	126.6	7.19–7.34 (*m*)	
2α	37.2	2.28 (1H, *dd*, *J* = 17.6, 7.8)	C-1, C-3, C-4	10,12	128.7	7.19–7.34 (*m*)	
2β		2.64 (1H, *dd*, *J* = 17.6, 5.5)	C-1	11	128.0	7.19–7.34 (*m*)	
3	54.1	2.98 (1H, *m*)		14α	82.9	4.13 (1H, *d*, *J* = 9.2)	C-15, C-3, C-5
4	35.6	1.74 (2H, *m*)	C-3, C-5	14β		4.64 (1H, *d*, *J* = 9.2)	C-3, C-5
5	77.3	4.17 (1H, *m*)		15α	58.9	3.82 (1H, *d*, *J* = 8.7)	C-1, C-3
6	128.3	6.20 (1H, *dd*, *J* = 16.0, 6.0)	C-5, C-8	15β		4.34 (1H, *dd*, *J* = 8.7, 2.7)	
7	131.3	6.63 (1H, *d*, *J* = 16.0)	C-5, C-9, C-13	N-H	-	6.41 (NH, *br s*)	
8	136.4	-					

**Figure 2:**
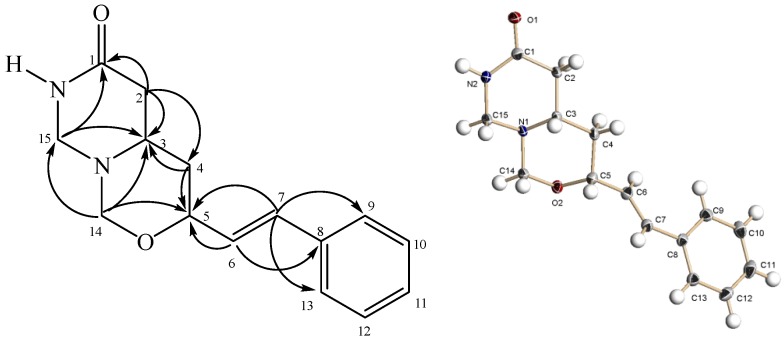
HMBC correlations and ORTEP diagram of **4**.

The relative stereochemistry of **4** was established with the aid of the NOESY spectrum. H-15α which is *axially* oriented showed a NOESY correlation with H-3, which in turn showed correlation with H-5, therefore suggesting that both H-3 and H-5 assume an *α-*spatial orientation. The structure and relative stereochemistry was further confirmed using single-crystal X-ray diffraction analysis CCDC 912021 contains the supplementary crystallographic data for this paper. These data can be obtained free of charge via www.ccdc.cam.ac.uk/conts/retrieving.html (or from the CCDC, 12 Union Road, Cambridge CB2 1EZ, UK; fax: +44 1223 336033; e-mail: deposit@ccdc.cam.ac.uk). An ORTEP drawing of **4** is shown in Figure 2. 

Tapisoidin (**5**) was obtained as pale yellowish amorphous powder, 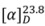
−25.9° (c 0.0058, CH_2_Cl_2_). Its molecular formula was determined to be C_18_H_17_O_4_N by ESI-TOF-MS (*m/z* 334.1074, [M+Na]^+^; calc. 334.1055). The IR spectrum displayed a band at 1715 cm^−1^ representing the C=O group of the molecule. The UV spectrum (λ_max_ 209, 251, 277 and 322 nm) indicated the presence of a basic aristolactam alkaloid structure [11]. The ^1^H-NMR spectrum (Table 4) showed signals for four adjacent aromatic protons at δ 8.35 (H-5, *dd*, *J* = 7.9, 1.4), δ 7.40 (H-6, *m*), δ 7.44 (H-7, *m*) and δ 7.35 (H-8, *dd*, *J* = 7.9, 1.4). Signals for three methyl groups at δ 3.96, δ 3.89 and δ 4.00 were assigned to the methoxyl groups at N-11, C-3 and C-4, respectively. The positions of the methoxyl groups were established from the NOESY and HMBC spectra. In the NOESY spectrum, the methyl protons of the methoxyl group at C-3 showed correlations with H-2. In the HMBC spectrum, the proton signal at δ 7.23 (H-2) correlated with the carbons signal at δ 59.7 (3-OCH_3_) and δ 56.0 (4-OCH_3_), thereby confirmed the assignments of the methoxyl groups.

**Table 4:** ^1^H-NMR, ^13^C-NMR and HMBC (400 MHz) data of **5** (CDCl_3_, δ in ppm, *J* in Hz).

Atom no.	δ ^13^C	δ^1^H	HMBC	Atom no.	δ ^13^C	δ^1^H	HMBC
1	123.1	-		8a	131.3	-	
2	105.9	7.23 (1H, *s*)	C-3, C-4, C-10a, C-12	9α	34.8	2.73 (1H, *t*, *J* = 13.8)	C-8a, C-10, C-10a
3	150.5	-		9β		3.49 (1H, *dd*, *J* = 13.8, 6.0)	C-10a
4	155.7	-		10	57.3	4.60 (1H, *dd*, *J* = 13.8, 6.0)	C-10a
4a	123.8	-		10a	134.4	-	
4b	135.1	-		12	167.9	-	
5	127.7	8.35 (1H, *dd*, *J* = 7.9, 1.4)	C-4a, C-4b	3-OCH_3_	59.7	3.89 (3H, *s*)	C-3
6	127.9	7.40 (1H, *dd*, *J* = 7.9, 1.4)	C-7	4-OCH_3_	56.0	4.00 (3H, *s*)	C-4
7	130.0	7.44 (1H, *dd*, *J* = 7.9, 1.4)	C-6, C-8a	N-OCH_3_	63.7	3.96 (3H, *s*)	
8	128.7	7.35 (1H, *dd*, *J* = 7.9, 1.4)	C-6, C-4b				

The ^1^H NMR spectrum of **5** also showed signals at δ 2.73 (H-9α, *t*, *J* = 13.8), δ 3.49 (H-9β, *dd*, *J* = 14.2, 6.0), δ 4.60 (H-10, *dd*, *J* = 13.8, 6.0), indicating that C-9 and C-10 are hydrogenated. To the knowledge of the authors, this is the first occurrence of a 9,10-dihydroaristolactam alkaloid. The ^13^C-NMR spectrum (Table 4) showed the presence of eighteen carbons; three methyl, one methylene, six methine, seven quaternary and one carbonyl. The carbonyl carbon (C-12) resonated at δ 167.9. Thus, taking into consideration all the NMR data and analyses, the structure of tapisoidin (**5**) was elucidated as illustrated in Figure 1. Identification of the known compounds was done by comparison of ^1^H- and ^13^C-NMR data with reported values [13,14,15,16,17,18].

### 2.2. Bioactivity

The result of cytotoxicity tests on the hexane, CH_2_Cl_2_ and MeOH crudes of *G. tapisoides* against lung cancer (A549), breast cancer (MCF-7) and prostate cancer (DU-145) cell lines are shown in Table 5. Hexane and CH_2_Cl_2_crude extractsexhibited cytotoxicity against the three cancer cell lines.

**Table 5:** Cytotoxicity of crude extracts from *G. tapisoides* against selected cancer cells at 100 µg/mL.

Extracts	Cell Viability (%)		
	A549	MCF-7	DU-145
Hexane	2.9	6.2	9.4
CH_2_Cl_2_	2.9	6.4	8.3
Methanol	87.2	94.8	82.3

Among the isolated compounds, compounds **1**–**6** were evaluated for their cytotoxicity (Table 6). Onlycompound **6** exhibited cytotoxic activity against all the eight cell lines. No cytotoxic activity was found for compounds **1**–**5**, most probably due tolack of pharmacophoric groups responsible for the high antiproliferative activity [3].

**Table 6:** Biological activitiesof theisolated compounds (IC_50_, µM, mean ± s.d., n = 3).

Cmpd.	Antioxidant activity	Cytotoxicity							
		A549 ^1^	DU-145 ^2^	SK-MEL-5 ^3^	BxPC-3 ^4^	Hep G2 ^5^	HT-29 ^6^	MCF-7 ^7^	MDA-MB-231 ^8^
1	0.328	>150	>150	>150	>150	>150	>150	>150	>150
2	0.207	>150	>150	>150	>150	>150	>150	>150	>150
3	1.748	>150	>150	>150	>150	>150	>150	>150	>150
4	0.252	>150	>150	>150	>150	>150	>150	>150	>150
5	0.772	>150	>150	>150	>150	>150	>150	>150	>150
6	2.024	107.62 ± 4.67	71.79 ± 1.61	100.14 ± 11.84	130.48 ± 7.69	128.73 ± 1.81	64.17 ± 5.60	120.37 ± 11.11	>150
Cisplatin	-	37.37 ± 3.00	15.18 ± 0.49	31.82 ± 0.23	20.10 ± 1.21	22.07 ± 0.64	77.24 ± 3.23	91.49 ± 6.54	276.53 ± 1.29
Ascorbic acid	0.075	-	-	-	-	-	-	-	-

^1 ^human lung carcinoma; ^2 ^human prostate carcinoma; ^3^skin cancer cell line; ^4 ^human pancreatic carcinoma; ^5^ human liver carcinoma;^ 6 ^human colon carcinoma; ^7,8 ^human breast carcinomas.

Compound **6** is the most potentagainstthe colon cancer cell line (HT-29). It exhibitedan IC_50_ of 64.17 ± 5.60 µM, which is comparable to that of cisplatin (77.24 ± 3.23 µM). Therefore the cytotoxicity of both hexane and dichloromethane extracts may be attributed to the presence of compound **6**, which has been previously reported to exhibit cytotoxicity against variouscancer cell lines [13,19]. In addition, reports have shown that the antiproliferative activity of **6** is selective for cancer cell lines with no significant cytotoxicity toward non-malignant cells [13,20].

Since free radicals are associated with DNA damage and protein modifications, including apoptotic modulators which could lead to carcinogenesis [21], we have also evaluated the antioxidant activity using a DPPH radical scavenging assay. Compound **2** gave the highest antioxidant activity, with an IC_50_ of 0.207 µM, followed by compounds **4** (IC_50_ = 0.252 μM) and **1** (IC_50_ = 0.328 μM). The high antioxidant effect of compounds **2**, **4** and **1** may be attributed to the presence of the hydroxyl group adjacent to the conjugated double bond that could donate electron to the DPPH free radical. Carotenes and xanthophyll which possess hydroxyl groups and conjugated double bonds have been reported to show high antioxidant activity [22,23]. Compounds **3** (IC_50_ = 1.748 μM), **5 **(IC_50_ = 0.772 μM) and **6** (IC_50_ = 2.024 μM) which lack hydroxyl groups in their structures showed very low antioxidant activity.

## 3. Experimental

### 3.1. General

Melting points were determined by a Fargo MD-1D melting point apparatus. The specific rotations were measured on a Jasco P-1020 polarimeter. UV spectra were recorded on a Shimadzu 1650 PC ultraviolet-visible spectrometer, and IR spectra on a Perkin Elmer Spectrum 400 FT-IR/FT-FIR spectrometer. 1D and 2D NMR spectra were measured with a JEOL ECA 400 spectrometer (400 MHz). The mass spectra were obtained on an Agilent Technologies 6530 Accurate-Mass-Q-TOF LC/MS. Column chromatography was performed on silica gel Merck 60 (230-400 mesh). X-ray data collection was obtained from a Bruker *APEX2* unit; cell refinement: *SMART*; data reduction: *SAINT*; program(s) used to solve the structure: *SHELXTL*; program(s) used to refine structure: *SHELXTL*.

### 3.2. Plant Material

The stem bark of *G. tapisoides* was collected from Sarawak and identified by Prof. Fasihuddin bin Ahmad, Faculty of Resource Science and Technology, Universiti Malaysia Sarawak. A voucher specimen (HUMS 000108) is deposited at the Herbarium of Universiti Malaysia Sarawak, Kota Samarahan, Sarawak, Malaysia.

### 3.3. Extraction and Isolation

The dried and milled stem barks of *G. tapisoides* (1.5 kg) were extracted successively with hexane, dichloromethane (CH_2_Cl_2_) and MeOH (15 L) to give 25 g of hexane extract, 43 g of CH_2_Cl_2_ extract and 24 g of MeOH extract, respectively, after removal of the solvents. The hexane extract (10 g) was subjected to silica gel column chromatography (CC), using hexane and CH_2_Cl_2_ with increasing polarity to give **6** (2.5 g). Then, the CH_2_Cl_2_ extract (20 g) was fractionated using CC with a gradient system of hexane/CH_2_Cl_2_ and CH_2_Cl_2_/MeOH to afford 11 fractions (F1-F11). F8 (CH_2_Cl_2_/MeOH, 90:10) was separated on a column of silica gel using CH_2_Cl_2_/MeOH (97:3) as the eluent to give **1** (12.3 mg). F9 (CH_2_Cl_2_/MeOH, 80:20) was further chromatographed on a silica gel column eluting with 100% CH_2_Cl_2_ to give **2**. F10 (CH_2_Cl_2_/MeOH, 50:50) was then purified by CC, using CH_2_Cl_2_/MeOH (99:1) to yield **3** (14.8 mg) and CH_2_Cl_2_/MeOH (96:4) to yield **4** (18.9 mg). F6 (CH_2_Cl_2_/MeOH, 98:2) was applied to a column of silica gel, eluted with CH_2_Cl_2_/MeOH (98:2) gave **5** (3.2 mg). F3 (Hex/CH_2_Cl_2_, 50:50) was further purified by silica gel CC using hexane/CH_2_Cl_2_ (50:50) as eluting solvent to afford **6** (1.3 g). F6 was subjected to silica gel CC, using 100% CH_2_Cl_2_ as eluent to yield **7** (4.8 mg). F7 (CH_2_Cl_2_/MeOH, 95:5) was separated on column of silica gel using CH_2_Cl_2_/MeOH (95:5) as developing solvent to yield **8** (1.1 g). Compounds **9** (1.2 mg), **10** (4.4 mg) and **11** (4.0 mg) were obtained from further purification of F6 (CH_2_Cl_2_/MeOH, 98:2) by CC using silica gel with a solvent system of CH_2_Cl_2_/MeOH (98:2).

### 3.4. Cytotoxicity Assay

Cytotoxicity of the compounds were evaluated against eight types of cancer cell lines; lung (A549), prostate (DU-145), skin (SK-MEL-5), pancreatic (BxPC-3), liver (Hep G2), colon (HT-29), breast cancer (MCF-7), and (MDA-MB-231). Cell lines were cultured in Dulbecco’s Modified Eagle medium (DMEM) with 10% foetal bovine serum. Cells were plated into 96-well microplates and 24 hour later, 100 µL of samples and cisplatin standard were introduced in triplicate. Cells were further incubated for 48 h and cell viability was determined using the MTS reagent (Promega Corp., Madison, WI, USA). Microplates were returned to the incubator for 1 h and absorbance was read on a microplate reader at 490 nm (Infinite 200, Tecan, Männedorf, Swizerland). The concentration required causing 50% cell death (IC_50_) by the samples and drug standards were determined using dose-response curves in Prism 5.02 software [24].

### 3.5. Antioxidant Assay

The DPPH antioxidant assay was determined as described by Shimada *et al.* [25]. Briefly, 0.1 mM DPPH (1 mL) dissolved in ethanol was added to an ethanol solution (3 mL) of the tested compound at different concentrations (0, 50, 100, 150, 200 µg/mL). An equal volume of ethanol was added in the control test. The mixture was shaken vigorously and allowed to stand at room temperature for 30 min. Then the absorbance at 517 nm was measured with a UV–VIS spectrophotometer. The percentage of scavenging of DPPH was calculated using the following equation:

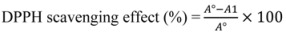

where A^o^ is the absorbance of the control reaction and A1 is the absorbance in the presence of the sample.

## 4. Conclusions

The CH_2_Cl_2_ extract of the stem bark of *G. tapisoides* yielded eleven compounds, of which five are new;goniomicins A-D (compounds **1**–**4**), and tapisoidin (**5**). The latter, tapisoidin (**5**), represents the first report of the occurence of a 9,10-dihydroaristolactam alkaloid. From this study, the compound responsible for the cytotoxicity of the extracts is goniothalamin (**6**), which showed the highest potency against the colon cancer cell line (HT-29) withan IC_50_ of 64.17 ± 5.60 µM. Goniomicin A (**1**), goniomicin B (**2**) and goniomicin D (**4**) displayed high antioxidant activity, which may be due to the presence of conjugated hydroxyl groups that could donate electrons to scavenge radicals [22,23].
